# The Clinical and Genetic Features in Chinese Children With Steroid-Resistant or Early-Onset Nephrotic Syndrome: A Multicenter Cohort Study

**DOI:** 10.3389/fmed.2022.885178

**Published:** 2022-06-09

**Authors:** Xiujuan Zhu, Yanqin Zhang, Zihua Yu, Li Yu, Wenyan Huang, Shuzhen Sun, Yingjie Li, Mo Wang, Yongzhen Li, Liangzhong Sun, Qing Yang, Fang Deng, Xiaoshan Shao, Ling Liu, Cuihua Liu, Yuanhan Qin, Shipin Feng, Hongtao Zhu, Fang Yang, Weimin Zheng, Wanqi Zheng, Rirong Zhong, Ling Hou, Jianhua Mao, Fang Wang, Jie Ding

**Affiliations:** ^1^Department of Nephrology, The Children Hospital of Zhejiang University School of Medicine, Hangzhou, China; ^2^Department of Pediatrics, Peking University First Hospital, Beijing, China; ^3^Department of Pediatrics, Fuzong Clinical Medical College, Fujian Medical University, Fuzhou, China; ^4^Department of Pediatrics, Guangzhou First People's Hospital, Guangzhou, China; ^5^Department of Nephrology and Rheumatology, Shanghai Children's Hospital, Shanghai Jiaotong University, Shanghai, China; ^6^Department of Pediatric Nephrology and Rheumatism and Immunology, Shandong Provincial Hospital Affiliated to Shandong University, Jinan, China; ^7^Guangzhou Women and Children's Medical Center, Guangzhou, China; ^8^Department of Nephrology, Ministry of Education Key Laboratory of Child Development and Disorders, Chongqing Key Laboratory of Pediatrics, National Clinical Research Center for Child Health and Disorders, China International Science and Technology Cooperation Base of Child Development and Critical Disorders, Children's Hospital of Chongqing Medical University, Chongqing, China; ^9^Department of Pediatrics, The Second Xiangya Hospital, Central South University, Changsha, China; ^10^Department of Pediatrics, Nanfang Hospital, Southern Medical University, Guangzhou, China; ^11^Department of Nephrology, The Second Affiliated Hospital and Yuying Children's Hospital of Wenzhou Medical University, Wenzhou, China; ^12^Department of Nephrology, Anhui Provincial Children's Hospital, Hefei, China; ^13^Department of Nephrology and Immunization, Guiyang Maternal and Child Health Care Hospital, Guiyang, China; ^14^Department of Nephrology and Rheumatology, Children's Hospital of Hebei Province, Shijiazhuang, China; ^15^Department of Nephrology and Rheumatology, Children's Hospital Affiliated to Zhengzhou University, Henan Children's Hospital, Zhengzhou Children's Hospital, Zhengzhou, China; ^16^Zhengzhou Key Laboratory of Pediatric Kidney Disease Research, Zhengzhou, China; ^17^Department of Pediatrics, The First Hospital of Guangxi Medical University, Nanning, China; ^18^Chengdu Women's and Children's Central Hospital, School of Medicine, University of Electronic Science and Technology of China, Chengdu, China; ^19^Department of Pediatrics, The First Affiliated Hospital of Xinjiang Medical University, Urumqi, China; ^20^Department of Pediatrics, The First Affiliated Hospital of Jinan University, Guangzhou, China; ^21^Department of Nephrology, Jiangxi Provincial Children's Hospital, The Affiliated Children's Hospital Nanchang University, Nanchang, China; ^22^Department of Pediatrics, The Second Hospital of Dalian Medical University, Dalian, China; ^23^Department of Pediatrics, Fujian Provincial Hospital, Fuzhou, China; ^24^Department of Pediatrics, Shengjing Hospital of China Medical University, Shenyang, China

**Keywords:** steroid-resistant nephrotic syndrome, genetic testing, phenotype, prognosis, children

## Abstract

Steroid-resistant nephrotic syndrome (SRNS) is one of the major causes of end-stage kidney disease (ESKD) in children and young adults. For approximately 30% of children with SRNS results from a genetic cause. In this study, genotype-phenotype correlations in a cohort of 283 pediatric patients with SRNS or early-onset NS (nephrotic syndrome presenting within the first year of life) from 23 major pediatric nephrology centers in China were analyzed. All patients were performed with next-generation sequencing and Sanger sequencing. The overall mutation detection rate was 37.5% (106 of 283 patients). WT1 was the most frequently detected mutation, followed by NPHS1, NPHS2, and ADCK4, and these four major causative genes (WT1, NPHS1, NPHS2, and ADCK4) account for 73.6% of patients with monogenic SRNS. Thirteen of 106 individuals (12.3%) carried mutations in ADCK4 that function within the coenzyme Q10 biosynthesis pathway. In the higher frequently ADCK4-related SRNS, two mutations, c.737G>A (p.S246N) and c.748G>C (p.D250H), were the most prevalent. Our study provides not only definitive diagnosis but also facilitate available targeted treatment for SRNS, and prediction of prognosis and renal outcome. Our indications for genetic testing are patients with FSGS, initial SRNS, cases of positive family history or those with extra-renal manifestations.

## Introduction

Idiopathic nephrotic syndrome (INS) is characterized by a group of symptoms: massive proteinuria, hypoalbuminemia, edema, and hyperlipidemia (1, 2). Most pediatric cases respond well to steroids, and the long-term prognosis is favorable (3). However, about 10–20% of children who do not show complete remission of proteinuria following 4–8 weeks treatment with corticosteroids are considered to have steroid-resistant nephrotic syndrome (SRNS), with high risk of end-stage kidney disease (3, 4). Kidney biopsy of SRNS shows minimal change disease (MCD) or focal segmental glomerulosclerosis (FSGS) in majority of cases (3, 5). For ~30% of children with SRNS, the condition results from a genetic cause, and who will not achieve remission after treatment with steroids and/or immunosuppression (6); and identification of these causative genes has provided fundamental insights into the pathogenesis of SRNS (3, 7–10).

Recently, increasing number of monogenic SRNS has been reported worldwide. To date, more than 60 monogenic causes of SRNS/FSGS have been identified, and novel causative genes are continually being discovered (3, 8, 11–13). Genetic testing for the children with initial SRNS, cases of positive family history with proteinuria and those with extra-renal manifestations is recommended, according to IPNA (International Pediatric Nephrology Association) clinical practice recommendations for the diagnosis and management of children with SRNS (14). In addition, most patients with early onset nephrotic syndrome presenting within the first year of life are caused by monogenic defects and resistant to immunosuppressive therapy, thus this condition is also an indication of genetic screening. The genetic testing about SRNS has been reported in America, Europe, and Asia such as in China, Korea, Japan and India (12, 13, 15–17). However, the high heterogeneity of genetic SRNS highlights the value of thoroughly delineating the correlations between genotype and phenotype based on large cohorts of patients with different ethnicities, molecular diagnosis and longitudinal follow-up. In this study we aimed to expand the genotypes of SRNS or early-onset NS in China and investigate potential correlations between genotype and phenotypes.

## Materials and Methods

### Patients

Pediatric patients with SRNS or early-onset NS, undergoing next-generation sequencing analysis, from 23 major pediatric nephrology centers in China, recorded in an on-line registry of pediatric hereditary kidney diseases in China (http://chkd.tiamal.com/, set up in 2012) from January 1, 2018 to December 31, 2020 were recruited. All patients had fully developed nephrotic syndrome (NS) (24 h urinary protein excretion ≥50 mg/kg or urinary protein creatinine ratio ≥ 2 g/g plus serum albumin <30 g/L). SRNS is consist of resistance to conventional daily oral 2 mg/kg prednisone (maximum dose 60 mg/day) therapy either at the initial presentation (initial steroid non-responders) or during follow-up (late steroid non-responders). An initial non-responder is defined as “failure to achieve complete remission after 4–8 weeks of corticosteroid therapy”, and a late non-responder is defined as “persistent proteinuria during 4 or more weeks of corticosteroids following one or more remissions” (18). Early-onset nephrotic syndrome is an uncommon disorder with onset of the nephrotic syndrome presenting within the first year of life. Demographics, clinical presentations at first visit to the participating centers, response to immunosuppressive agent, kidney biopsy information if performed, the last follow-up data, family history, parental consanguinity and genetic data were collected. The age-adjustment of serum creatinine concentrations based Chronic Kidney Disease Epidemiology Collaboration equation was used to estimate the glomerular filtration rate (19). When the patients were younger than 2 years, kidney dysfunction was defined as serum creatinine increasing >30% from the upper reference limits related to age and gender (20). For evaluation of renal outcomes, the primary end point included a set of major morbidity events such as reaching ESKD, renal replacement therapy (RRT, hemodialysis, peritoneal dialysis, kidney transplantation), and mortality from renal cause.

Genomic DNA was isolated from blood lymphocyte in all participants and subjected to exome capture using Agilent's SureSelect human all exon kit V5 and NimbleGen technology followed by next generation sequencing on the Illumina HiSeq 2500 platform. The genetic test results which were detected by next-generation sequencing and Sanger sequencing in the DNA diagnostic laboratories were collected and classified according to the American College of Medical Genetics and Genomics (**ACMG**) guidelines (21). The patients harbored pathogenic or likely pathogenic sequence variants plus consistent with the reported inheritance were considered establishing a genetic diagnosis.

The procedures were approved by the ethics committees of the 23 centers. The informed consent was obtained from the patients or their family members.

### Statistical Analysis

To determine significant differences between groups with or without pathogenic variants, categorical variables were analyzed using the chi-square test or Fisher's exact test, and continuous variables were compared using the *t*-test or Mann–Whitney *U*-test. All values are reported as the median (interquartile range, IQR).

## Results

### Cohort Description

**Figure 1:**
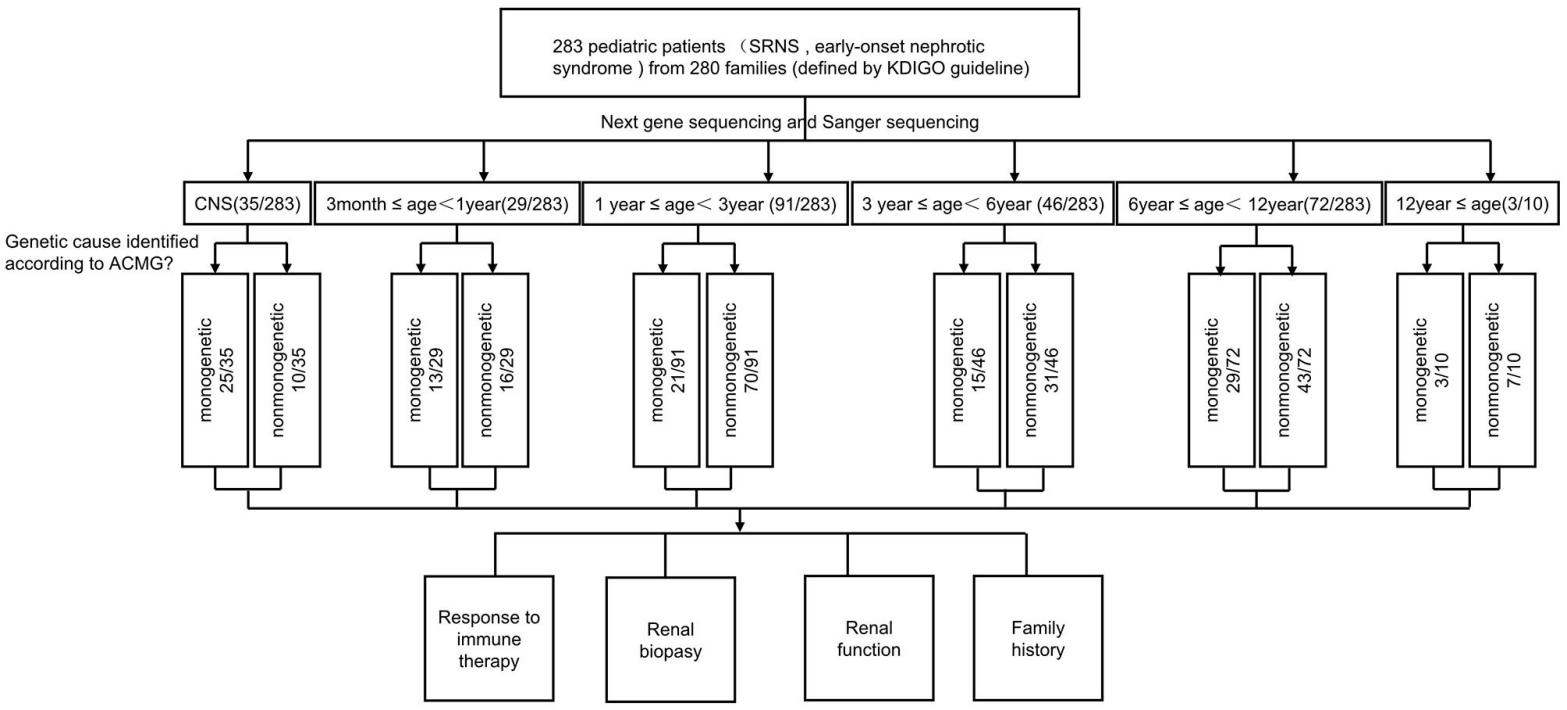
Flow chart of patients by phenotype at presentation, age, and genetic diagnosis.

Totally 283 pediatric patients (male: female = 151:132) from 280 families were recruited from the 23 major pediatric nephrology centers in China. All patients had fully developed early-onset NS or SRNS. Among the patients with available respective information, parental consanguinity was not reported, and 30 (10.6%) patients had a family history of proteinuria and/or renal failure (Table 1 and Figure 1).

**Table 1:** Genotype-phenotype correlations in pediatric patients with steroid-resistant nephrotic syndrome.

		**CNS**		**Infantile**		**1–2 years**		**3–5 years**		**6–12 years**		**≥12 years**		**Total patient**		***P*-value**
		***n =*** **35(12.4%)**		***n =*** **29 (10.2%)**		***n =*** **91 (32.2%)**		***n =*** **46 (16.3%)**		***n =*** **72 (25.4%)**		***n =*** **10 (3.5%)**		***n =*** **283**		
		**Mutation**	**Mutation**	**Mutation**	**Mutation**	**Mutation**	**Mutation**	**Mutation**	**Mutation**	**Mutation**	**Mutation**	**Mutation**	**Mutation**	**Mutation**	**Mutation**	
		**(−)**	**(+)**	**(−)**	**(+)**	**(−)**	**(+)**	**(−)**	**(+)**	**(−)**	**(+)**	**(−)**	**(+)**	**(−)**	**(+)**	
		***n =* 10**	***n =* 25**	***n =* 16**	***n =* 13**	***n =* 70**	***n =* 21**	***n =* 31**	***n =* 15**	***n =* 43**	***n =* 29**	***n =* 7**	***n =* 3**	***n =* 177**	***n =* 106**	
Sex	Male:female	7:3	15:10	5.11	7:6	42:28	8.13	16.15	8:7	29:14	11.18	2:5	1:2	101:76	50:56	
Response to steroid	No treatment	6	18	0	2	0	0	2	0	0	1	0	0	8 (4.5%)	21 (19.8%)	
	Initial non-respond	3	5	14	9	50	16	16	14	36	19	4	3	123 (69.5%)	66 (62.3%)	
	Late non-responder	0	0	2	0	7	0	7	0	4	1	2	0	22 (12.4%)	1 (0.9%)	
	Data unavailable	1	2	0	2	13	5	6	1	3	8	1	0	24 (13.6%)	18 (17.0%)	
Response to immune therapy	No treatment	7	22	9	8	11	9	7	4	9	12	1	1	44 (24.9%)	56 (51.9%)	
	Responder	0	1	1	0	28	1	12	0	19	0	3	0	63 (35.6%)	2 (1.9%)	
	Non-responder	3	2	6	5	31	11	12	11	15	17	3	2	70 (39.5%)	48 (46.2%)	<0.001^**a**^
Kidney biopsy	FSGS	1	1	5	3	18	6	16	9	19	15	1	3	60 (33.9%)	37 (34.9%)	<0.01^f^
	MCD	0	0	1	0	17	0	8	0	10	4	2	0	38 (21.5%)	4 (3.8%)	<0.01^f^
	MsPGN	0	1	0	2	9	3	4	0	2	1	1	0	16 (9%)	7 (6.6%)	
	Others	1	0	0	0	6	1	0	2	10	1	3	0	20 (11.3%)	4 (3.8%)	
	Not done	8	23	10	8	20	11	3	4	1	8	0	0	42 (23.7%)	54 (51.0%)	
Family history	Yes	1	1	3	2	6	4	3	1	3	6	0	1	16 (8.8%)	14 (13.2%)	
	No	8	24	12	10	60	17	27	14	39	22	7	2	153 (86.2%)	90 (84.9%)	<0.01^d^
	Data unavailable	1	0	1	1	4	0	1	0	1	1	0	0	8 (4.5%)	2 (1.8%)	
Extrarenal manifestations	Yes	4	8	2	5	5	4	5	6	7	3	0	0	23 (11.5%)	26 (24.5%)	
	No	6	17	14	8	65	17	26	9	36	26	7	4	154 (87%)	80 (75.5%)	<0.001^e^
Renal outcome at follow-up	Normal eGFR	1	5	7	3	31	5	11	8	16	7	2	0	68 (38.4%)	28 (26.4%)	
	CKD stages 2–4	0	0	1	1	3	0	6	1	7	5	2	2	19 (10.8%)	9 (8.5%)	<0.01^**b**^
	ESKD/RRT	0	0	1	3	7	10	6	4	6	11	0	0	20 (11.3%)	28 (26.4%)	<0.01^**c**^
	Mortality	4	8	2	3	5	3	0	1	0	1	0	0	11 (6.2%)	16 (15.1%)	
	Data unavailable	5	12	5	3	24	3	8	1	14	5	3	1	59 (33.3%)	25 (23.6%)	
Renal transplantation	Yes	0	0	0	1	3	7	0	0	1	6	0	0	4 (2.2%)	14 (13.2%)	
	No	6	20	11	10	34	12	18	11	37	19	4	2	110 (62.1%)	74 (70%)	
	Data unavailable	4	5	5	2	33	2	13	4	5	4	3	1	63 (35.6%)	18 (17.0%)	

a *respond to immune thearpy group vs. non-responders*.

b *normal eGFR vs. CKD stage 2–4*.

c *CKD stage 2–4 vs. groups(ESKD+Mortality)*.

d *groups with family history vs. groups without family history*.

e *groups with extrarenal manifestations vs. groups without extrarenal manifestations*.

f *FSGS vs. MCD*.

*CNS, congenital nephrotic syndrome; FSGS, focal segmental glomerulosclerosis; MCD, Minimal change disease; MsPGN, Mesangial proliferative glomerulonephritis; eGFR, estimated glomerular filtration rate; CKD, chronic kidney disease; ESKD, end-stage renal disease; RRT, Renal replacement therapy (hemodialysis, peritoneal dialysis, kidney transplantation)*.

### Phenotypes

The clinical features of 283 patients [including 92 cases reported from our previous study (13)] are summarized in Table 1. Among them, 35 (12.4%) patients showed congenital onset, 29 (10.2%) patients manifested as infantile onset, 91 (32.2%) at age of 1–2 years. The number decreased to 46 cases (16.3%) at age 3–5 years, 72 (25.4%) at age 6–12 years and 10 (3.5%) patients at age 12–18 years.

**Figure 2:**
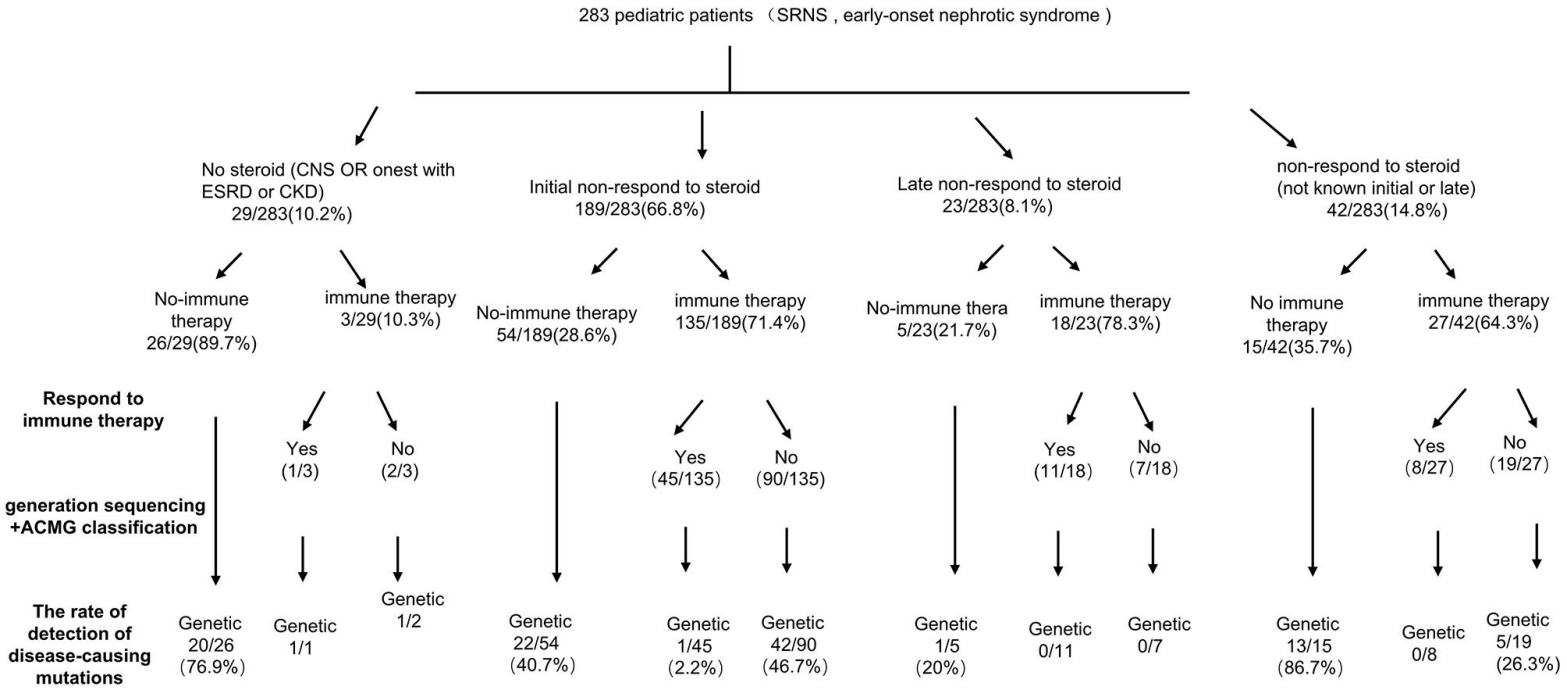
A precision medicine-based guide to investigation of SRNS.

Among 283 patients recruited in the present study, 29 patients were not given steroid therapy because they presented with congenital onset (*n* = 24), 4–12 months (*n* = 2), CKD (*n* = 1) and 2 patients deny steroid therapy. Then as described in Figure 2, conventional steroid therapy was administered to 254 patients and all showed steroid resistance, consist of initial SRNS (189 patients) and late SRNS (23 patients) and 42 patients of SRNS not known initial or secondary. Among the 254 patients, 74 patients were not given immune therapy, 64 patients showed response to immune therapy, and 116 patients showed no response to immune therapy. Of the patients who responded to immunotherapy whereas presented with initial/secondary SRNS or without steroid therapy, the genetic diagnosis was established in 3 children (Supplemental Table 1). Patient 9th had the compound heterozygous pathogenic NPHS1 mutation (c.2515delC, p.Q839 Rfs^*^8 and c.928G>A, p.D310 N), developed NS at age of 2 month, Kidney biopsy was not performed and was then only given tacrolimus treatment from age of onset, proteinuria decreased and complete remission of proteinuria was observed after one year, this patient had a normal renal function after a 2-year follow up. Patient 232th displayed with initial SRNS and was given tacrolimus treatment, complete remission of proteinuria was observed, compound heterozygous pathogenic NPHS2 mutation (c.370T>C, p.C124R and c.535-1G>T) was detected in this boy, with the renal biopsy revealing mild mesangial proliferative glomerulonephritis and renal function was normal after one-year follow up. Patient 57th, a 7-year-old boy, was admitted to hospital for proteinuria due to nephrotic syndrome, then displayed with late-SRNS. Renal biopsy revealed Lipoprotein glomerulopathy. DNA sequence studies revealed a heterozygous pathogenic missense mutation of ApoE (c.127C>T, p.R43C).

Renal biopsy was performed in 187 (66.1%) patients, which included 97 cases of FSGS, 42 cases of MCD, and 23 patients with mesangial proliferative glomerulonephritis (MsPGN). Renal biopsy was done in 85% cases aged older than 3 years, 66.3% aged 1–2 years, and 22.2% in infant, respectively. Genetic abnormalities were found in 37.4% of patients with FSGS, 30% of patients with MsPGN, and 9.3% of patients with MCD. FSGS counts for 71.5% in the patients with an identified genetic mutation, whereas only 44.4% in the patients without genetic mutation. MCD was seen in 3.7% of the patients with a genetic diagnosis (4/107), all manifested at age of 6–12 years old. Table 2 summarized the data on the genetic disease detection rate from the previous large cohort studies and compared to the present study (12, 15, 22).

**Table 2:** Age at onset, comparison with previous large studies.

	**The age of onset**	**Present study**	**Eujin Park (Korea)**	**China Nagano (Japan)**	**Carolin E**
Percent with causative mutation detectation	1–3 m	25/35 (71.4%)	27/35 (77.1%)	11/13 (84%)	163/235 (69.5%)
	3 m−1 y	13/29 (44.8%)	11/25 (44%)	8/15 (53%)	81/163 (49.6%)
	1–3 y	21/91 (23.1%)	40/114 (35%)^*^	23/89 (26%)	177/700 (25%)
	3–6 y	15/46 (32.6%)		6/36 (17%)	56/315 (17.7%)
	6–12 y	29/72 (40.3%)	15/36 (27.7%)	16/52 (31%)	19/176 (11%)
	12–18 y	3/10 (33.3%)	2/3 (66.7%)	3/15 (20%)	6/28 (21.4%)
CNS	NPHS1 (17/25)	WT1 (15/31)	NPHS1 (4/11)	NPHS1 (94/163)
	WT1 (3/25)	NPHS1 (11/31)	LAMB2 (4/11)	NPHS2 (25/163)
	NPHS2 (2/25)	COQ3 (3/31)	WT1 (2/11)	WT1 (20/163)
	LAMB2 (2 /25)	LAMB2 (2/31)	LAMA5 (1/11)	LAMB2 (13/163)
	ADCK4 (1/25)			

*CNS, congenital nephrotic syndrome*.

* *The percent of causative mutation detectation rate at age of oneset of 3–6 years*.

The median duration of follow-up from the onset was 2.1 years (IQR 1.2–3.4 years) in present study. 96 patients had maintained a normal estimated glomerular filtration rate (eGFR), 28 patients progressed to CKD stages 2–4, 48 patients progressed to ESKD and RRT, 27 patients end with mortality. Among the children who underwent mutation screening, the patients with an identified gene mutation are more likely to step into renal dysfunction: 9 patients (8.5%) with CKD, 28 patients (26.4%) with ESKD or RRT, and 16 (15.1%) patient end with mortality. Conversely in the patients not identified: 19 (10.7%) in patients with CKD stages 2–4, 20 (11.3%) in patients with ESKD or RRT and 11 patients (6.2%) end with mortality. SRNS children with identified causative genes showed worse long-term outcome than the patients without causative genetic mutation.

### Genotypes

The distribution of the detected causative genes was evaluated in Table 3. The overall detection rate of disease-causing mutations was 37.5% (106 of 283 patients). Among 106 patients with disease-causing mutations, 44 (41.5%) patients had AD mutations, 56 (52.8%) patients had AR mutations, and 6 (5.6%) patients had X-linked or mitochondrial mutations. WT1 was the most common causative gene (11.3%, 32 patients), followed by NPHS1 (6.4%, 18 patients), NPHS2 (5.3%, 15 patients), ADCK4 (4.6%, 13 patients), TRPC6 (2.1%, 6 patients) and others. Among these, mutations from 25 patients had been reported in our previous study (13).

**Table 3:** Genotype-phenotype correlations in pediatric patients with steroid-resistant nephrotic syndrome of AD mutation and AR mutation.

		**AD**					**AR**					
		**WT1**	**TRPC6**	**ACTN4**	**Others**	**Total**	**NPHS1**	**NPHS2**	**ADCK4**	**LAMB2**	**Others**	**Total**
		**(*n =* 32)**	**(*n =* 6)**	**(*n =* 3)**	**(*n =* 3)**	**(*n =* 44)**	**(*n =* 18)**	**(*n =* 15)**	**(*n =* 13)**	**(*n =* 3)**	**(*n =* 7)**	**(*n =* 56)**
Sex	Male:Female	8:24	4:2	1:2	2:1	15:29	14:4	9:6	4:9	0:3	5:2	32:24
Age of onset	1–3 m	3	0	0	0	3 (6.8%)	17	2	1	2	0	22 (39.3%)
	3 m−1 y	6	1	0	0	7 (15.9%)	0	3	1	1	1	6 (10.7%)
	1–2 y	9	2	0	0	11 (25%)	0	5	3	0	2	10 (17.9%)
	3–5 y	5	1	1	1	8 (18.2%)	1	0	2	0	2	5 (8.9%)
	6–12 y	7	2	2	2	13 (29.5%)	0	3	6	0	2	11 (19.6%)
	≥12 y	2	0	0	0	2 (4.5%)	0	2	0	0	0	2 (3.6%)
Kidney biopsy	FSGS	8	4	2	1	15 (34%)	1	6	5	1	5	18 (32.1%)
	MCD	1	0	0	0	1 (2.2%)	0	1	0	0	0	1 (1.8%)
	MsPGN	1	0	0	0	1 (2.2%)	1	3	1	0	0	5 (8.9%)
	Others	3	1	0	1	5 (11.3%)	0	0	0	0	0	0
	Not done	19	1	1	1	22 (50%)	16	5	7	2	2	32 (57.1%)
Renal outcome at follow-up	Normal eGFR	6	1	0	2	9 (20.5%)	4	8	3	0	3	18 (32.1%)
	CKD stages 2–4	4	0	0	0	4 (9.1%)	2	0	0	1	1	4 (7.1%)
	ESKD/RRT	9	5	2	1	17 (38.7%)	0	5	6	0	2	13 (23.2%)
	Mortality	8	0	1	0	9 (20.5%)	6	0	0	0	1	7 (12.5%)
	Data unavailable	5	0	0	0	5 (11.4%)	6	2	4	2	0	14 (25%)
Family history	Yes	2	0	0	1	3 (6.7%)	0	5	6	0	1	12 (21.4%)
	No	29	6	3	2	40 (91%)	17	10	7	2	5	31 (55.4%)
	Data unavailable	1	0	0	0	1 (2.2%)	1	0	0	1	1	3 (5.3%)
Extrarenal manifestations	Yes	12	0	0	0	12 (27.2%)	4	4	0	2	3	13 (23.2%)
	No	20	6	3	3	32 (72.7%)	14	11	15	1	4	43 (76.8%)
Renal transplantation	Yes	2	3	0	0	5 (11.4%)	0	3	2	0	2	9 (16.1%)
	No	24	2	3	3	32 (72.7%)	12	8	8	0	2	30 (53.6%)
	Data unavailable	6	1	0	0	7 (15.9%)	5	4	3	3	3	17 (30.3%)

*AD, autosomal dominant; AR, autosomal recessive. FSGS, focal segmental glomerulosclerosis; MCD, Minimal change disease; MsPGN, Mesangial proliferative glomerulonephritis; eGFR, estimated glomerular filtration rate; CKD, chronic kidney disease; ESKD, end-stage renal disease;RRT, Renal replacement therapy (hemodialysis, peritoneal dialysis, kidney transplantation)*.

### Genotype-Phenotype Correlations

To examine genotype-phenotype correlations, the 7 most frequently mutated genes (WT1, NPHS2, NPHS1, TRPC6, ACTN4, ADCK4 and LAMB2) were evaluated in Table 3. In the patient with AD mutations, 6.8% patients showed congenital onset, and 15.9% showed infantile onset. This fraction changed to 43.2% at age 1–5 years, 29.5% at age 6–12 years, and 4.5% at age 12–18 years. For the AR mutations, 39.3% patients showed congenital onset, and 10.7% showed infantile onset. This fraction decreased to 26.8% at age 1–5 years, 19.6% at age 6–12 years, and 3.6% at age 12–18 years. The distribution of causative genes within the first 3 months of life was as follows: NPHS1 (*n* = 17), WT1 (*n* = 3), NPHS2 (*n* = 2), LAMB2 (*n* = 2), ADCK4 (*n* = 1). Patients with AR mutations are more likely to display with congenital-onset SRNS, especially NPHS1, 17 (94.4%) onset at <3 months old.

FSGS was the most common pathological finding on renal biopsy in this cohort study, 34% for AD mutation and 32.1% for AR mutation. But in AR mutation, 32 (57.1%) biopsy was not performed most likely because of the high risk of percutaneous renal biopsy in patients with congenital-onset SRNS.

For WT1 mutations, 12 participants revealed non-truncating transcriptional variants and 20 participants revealed truncating mutation, 10 patients with non-truncating mutation show almost onset <3 years old (Table 4), 20 patients with truncating mutation show a wide distribution from 3 months to 12 years onset. 9 patients with truncating mutation end with ESKD/RRT or death. For the 18 NPHS1-related SRNS comprised of 17 CNS, all displayed with compound heterozygous mutation, 2 patients were lost to follow-up.

**Table 4:** Mutation screening results.

		**WT1**		**NPHS1**		**NPHS2**				**ADCK4**		
		**Heterozygous**		**Compound heterozygous**		**Hom mutation**		**Compound heterozygous**		**Hom mutation**	**Compound heterozygous**	
		**Non-truncating**	**Truncating**	**Truncating +truncating**	**Truncating +missense**	**Missense**	**Truncating**	**Truncating + truncating**	**Truncating + missense**	**Missense**	**Missense + missense**	**Truncating + missense**
		**(*n =* 12)**	**(*n =* 20)**	**(*n =* 9)**	**(*n =* 9)**	**(*n =* 2)**	**(*n =* 4)**	**(*n =* 5)**	**(*n =* 4)**	**(*n =* 6)**	**(*n =* 4)**	**(*n =* 3)**
Age of onset	1–3 m	2	1	9	8	0	0	1	1	1	0	0
	3 m−1 y	3	3	0	0	0	3	0	0	0	0	1
	1–2 y	5	4	0	0	0	1	2	2	2	1	0
	3–5 y	0	3	0	1	0	0	1	0	1	0	1
	6–12 y	1	6	0	0	1	0	1	1	2	3	1
	≥12 y	1	1	0	0	1	0	0	0	0	0	0
Renal Outcome at follow-up	Normal eGFR	1	5	2	2	1	2	3	2	2	1	0
	CKD stages 2–4	1	3	0	1	0	0	0	0	0	0	0
	ESKD/RRT	2	6	1	0	0	1	2	2	2	2	2
	Mortality	5	3	3	3	0	0	0	0	0	0	0
	Data unavailable	3	3	3	3	1	1	0	0	2	1	1

*CKD, chronic kidney disease; ESKD, end-stage kidney disease; RRT, Renal replacement therapy (hemodialysis, peritoneal dialysis, kidney transplantation)*.

For the NPHS2 mutations, patients carried truncating mutation showed onset at earlier age, 4 cases carried compound heterozygous mutation step into ESKD or was given renal replacement therapy.

**Figure 3:**
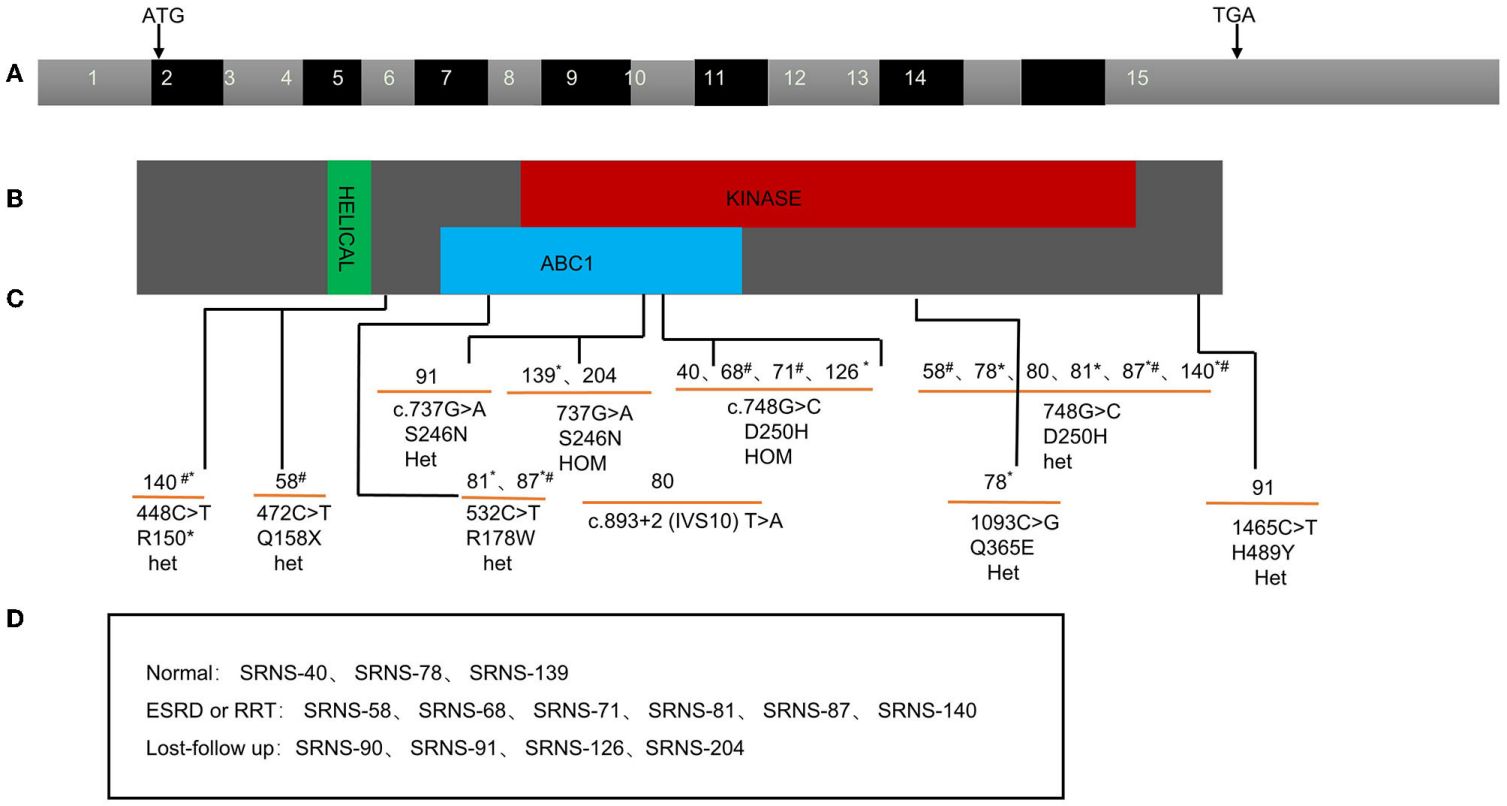
ADCK4 mutation found in pediatric SRNS in China. Exon capture revealed ADCK4 mutations as causing SRNS and CNS. **(A)** Exon structure of human ADCK4 cDNA which contains 15 exons. Positions of start codon (ATG) and of stop codon (TGA) are noted. **(B)** Domain structure of the ADCK4 protein. Extent of predicted domains, helical, ABC1, and kinase is depicted by colored bars, in relation to encoding exon position. **(C)** 13 different ADCK4 mutations with SRNS or or early-onset nephrotic syndrome. **(D)** The renal outcome of the patients during the follow-up. *Family history of proteinuria; ^**#**^Ending into ESKD or RRT during follow-up.

As for ADCK4 mutations, it can be detected in 13 (4.6%) in the SRNS. 6 patients present with homozygous and 7 patients displayed with compound heterozygous mutations. In present study, all 13 cases carried c.737G>A (p.S246N) and/or c.748G>C (p.D250H), 6 (42%) cases progressed to ESKD <1 years after diagnosis and 6 patients has family history of proteinuria (Figure 3).

Twenty-six patients (24.3%) with genetic-SRNS have extrarenal manifestations, mainly in WT1, SMARCAL1 and LAMB2. Thirty-seven percent of WT1-related SRNS showed genital abnormalities or a predisposition to Wilms tumor, 2 cases of SMARCAL1-related SRNS showed growth failure and poor cellular immunity. As to the three LAMB2-related SRNS, the age of onset was all younger than 1 years old, 2 of them with bilateral microcoria, presented with Pierson syndrome.

In our cohort study, five families of SRNS with disease-causing collagen COL4A5 mutations were identified. Extrarenal manifestations and family history of kidney disease were denied in these 5 patients. Light microscopy showed FSGS in 4 patients and MCD in 1 patient, while 1 patient revealed GBM lamellation, another showed segmental thickening in GBM.

A mutation in the CLCN5 gene was detected by exome sequencing in a family that had been defined as SRNS on clinical grounds. Patient 85th carried c.1942 C>T from his mother, and the light microscopy in this 10-year-old male with a CLCN5 mutation was MCD.

In family 48th, we detected a PAX2 splice mutation (c.70dupG). The patient presented with SRNS at the age of 4 year. Renal biopsy showed FSGS. The patient's father was diagnosed as ESKD.

## Discussion

In this cohort study, 283 children with SRNS or early-onset nephrotic syndrome were recruited from 23 pediatric nephrology centers in China, and 106 cases (37.5%) were identified with monogenic mutation. Specifically, the genetic finding of CNS was 71.4% in our study, the fraction of causative gene is only 23.1% at patients aged 1–3 years, and 35.8% at patients aged 3–18 years. Four major causative genes (WT1, NPHS1, NPHS2, and ADCK4) account for 73.6% of patients with monogenic SRNS. Among the 13 cases (12.3%) with ADCK4 mutation, 6 families with homozygous mutations and 7 families with compound heterozygous mutations, further, they all carried either **c.737G>A** (S246N) or **c.748G>C** (D250H) mutation.

Our previously cohort study enrolled 110 children with SRNS and 10 children with isolated proteinuria from 5 centers in China, and genetic etiology was identified in 28.3% patients and the most common mutated genes were ADCK4 (6.67%), NPHS1 (5.83%), WT1 (5.83%), and NPHS2 (3.33%), which was quite different from this study. In 2019, national cohort of children with renal disease from 13 different regions of China were recruited from 2014 to 2018, ADCK4, WT1 and NPHS1 were the top three commonly mutated genes in the SRNS group with mutation rates of 5.7, 5.4, and 2.8%, respectively (17). One possible explanation for this discrepancy is the geographical differences in our two series: two-thirds of the participants (80 of 120 cases) in our previous series came from Northern China, whereas more than 50% of the participants (157 of 283 cases) in this study came from Southern China. While in the study reported by Jia Rao, 89.9% of the participants come from Eastern China. In addition, more individuals of SRNS in the present study were recruited than before, which might be the second cause for discrepancy. Age at first disease manifestation present almost half between 1 and 6 years of age in our previous study and this study, 10.2% were at early infantile and 25% at age 6–12 years in this study, 15% at early infantile and 28.3% at age 6–12 years in our previous cohort, which might be the third cause.

The genetic detection rate of CNS was 71.4% in our study. The distribution of causative genes of CNS was NPHS1, WT1, NPHS2, LAMB2 and ADCK4, respectively. According the study reported by Eujin Park (15) in 2020, WT1 was the most commonly mutated gene in CNS, followed by NPHS1, COQ3 and LAMB2. While in the study by Nagano C from Japan (23), NPHS1, LAMB2 and WT1 was the most commonly gene detected in CNS. So, the genetic detection rate and the leading genes in CNS vary among different countries and district worldwide. In present study, 11 CNS patients were treated with steroid but no response happened, therefore, identification of the causative mutation may avoid unnecessary initiation or extension of steroid treatment.

The fraction of causative gene is 59.3% in in the first year of life, and 26.3% at patients aged 2–6 years, 39.1% at age 7–18 years. In 2020, in the study reported by Eujin Park (15), 291 Korean pediatric patients with SRNS/FSGS were analyzed, the mutation detection rate onset at age 6–18 years is 43.5%. however, In the Caroline E's study (12), an international cohort of 1783 families were included, disease-causing mutation in 61.3% of children in the first year of life. This fraction decreased to approximately 25% at age 2–5 years, to 12.3% at age 6 and older groups, these participants did not include patients from Russia, China, sub-Saharan Africa, or Pacific Rim countries. The difference between the studies may be due, at least in part, to differences in geographical distribution.

The mortality rate is 33% in the patients with NPHS1 mutation, whereas 11.1% (10/90) in the groups with other gene mutation in our present study and 16% (6/37) of the children with NPHS1 mutations in a nationwide retrospective study conducted by Bérody S (24). Children who had *NPHS2* heterozygous mutations had significantly lower renal survival than those of children with homozygous mutations, well in line with previous cohort studies (25). Of cases with ADCK4 mutation, 46.1% presented with ESRD/RRT, compared with 40% of WT1 and 33.3% of NPHS2 cases, whereas 38.5% of ADCK4 disease progressed to ESRD, compared with 15.6% of WT1 and 2.9% of NPHS2 cases in a study of 534 consecutive SRNS cases (26).

In our study, 2 monogenic SRNS cases (1 for NPHS1 and 1 for NPHS2 mutation) responded fully to tacrolimus. It was reported that SRNS with WT1 mutation were more likely to respond to Calcineurin inhibitors (CNIs), followed by PLCE1, NPHS1, NPHS2 (27). It seems that CNIs can ameliorate proteinuria by affecting podocyte proteins, instead of immune mechanism in monogenetic SRNS. Several studies reported non-immunomodulatory mechanisms of CNIs on renal protection. Faul et al. have reported that CsA blocks the calcineurin-mediated dephosphorylation of synaptopodin, which is easy to be degraded and participated in the podocyte injury and proteinuria (28). Treatment with tacrolimus can suppress the redistribution of Nephrin, Podocin and other slit diaphragm components and then ameliorate proteinuria (29). However, there is still a far way to look for effective therapy for genetic kidney disease.

Compared with studies from other countries, one of the most striking findings in this study was the high prevalence of ADCK4 mutations, as identified in 12.3% of patients with monogenic SRNS, and in 4.6% of all SRNS in children. Similar with our study, 20 patients (5.8%) with biallelic mutations of ADCK4 was screened for in the Chinese children with SRNS, non-nephrotic proteinuria, or CKD of unknown origin by Raojia et al. in 2020 (30), two mutations, namely c.737G>A (p.S246N) and c.748G>C (p.D250H), were the most prevalent in these two studies. In the study by Eujin Park (15) in 2020, 291 Korean children with SRNS or FSGS was recruited, ADCK4 accounts 6.3% of the patients with mutations and 6 patients carried S246N mutation. In 2020, A total of 230 patients (22) (CNS, INS, SRNS, FSGS, or asymptomatic proteinuria with likely genetic disease) in Japan were included. ADCK4 accounts 2.4% of the patients with mutations, and these four cases showed compound heterozygous mutations and all carried S246N mutation. On the contrary, mutations in ADCK4 accounted for only 0.3% of patients in the PodoNet Registry study (31), while 1655 patients were registered and underwent comprehensive screening for a panel of 31 podocyte genes in 2015. ADCK4 mutations were found in 26 patients from 12 families (1.9%) from France, Turkey and Germany, c.1339dupG (p.E447Gfs^*^10) and c.532C>T (p.R178W) were the most prevalent mutation (26).

According to literature, ADCK4-related nephropathy usually presents in adolescence (median age, 14.1 years) (26). In our study, most ADCK4-related SRNS presented from 1 to 12 years old (IQR 2.3–7 years), and one case showed CNS, significantly younger at time of diagnosis. Furthermore, 6 cases progressed to ESKD <1 year after diagnosis. The fact that all 13 cases of our study carried S246N and D250H mutation, may prompt us to find more precise diagnosis and therapy to ADCK4-related SRNS.

In our overall cohort, 7 of 106 (6.6%) cases could be explained as phenocopies. 5 cases were genetically proven Alport syndrome (AS) that presented as SRNS clinically at onset without family history of hematuria and proteinuria. Among them, 4 cases proved to be FSGS and 1 case with MCD on renal biopsy. It is reported that, rare variants in COL4A3 and COL4A4 may be disease-causing in the patients with familial FSGS (32). In our study, variants in COL4A4 may be disease-causing, the symptoms of AS are atypical at early stage to be neglected and SRNS occurred due to primary GBM defect. It is known that mutations in LAMB2 can cause FSGS, (33) the GBM provide a mechanical foothold for its adjacent cells such as podocyte, LAMB2 and COL4A4 are the components of the GBM, the mutation of COL4A4 may cause glomerular disease through the same or different functional pathway, some mechanism study need to be further studied. One patient in present study was genetically proved Dent disease displaying clinical feature as SRNS. Mutations in CLCN5, which was encodes a voltage-gated chloride ion channel in the renal tubule, are the major cause of Dent disease. More than 50% patients with Dent disease will present with nephrotic-range proteinuria, which is the important reason for misdiagnosis as SRNS. One mutation in the CLCN5 gene (c.2000delC) was reported in a family of SRNS by the PodoNet cohort (34), and our case illustrated similar phenotypic traits at all (35). To our knowledge, 3 different mechanisms might explain patients with phenocopies: one for misdiagnosis: clinical or genetic misdiagnosis; the second for concomitant of two different diseases; the third one is the real phenocopy as causality was not established between certain phenotypes and genotypes. After all, phenocopy is common, and it should be careful to define the relation between phenotype and genotype in children with monogenic SRNS.

In 2020, IPNA clinical practice recommendations for the diagnosis and management of children with steroid-resistant nephrotic syndrome was published (14), and genetic testing is recommended for patients with initial SRNS, cases of positive family history and those with extra-renal manifestations, but not for patients with secondary steroid resistance. In the present study, 23 patients showed secondary steroid resistance. Among them, only 1 patient was identified carrying pathogenic mutation of ApoE. In our study, the mutation detection rate was higher in patients with FSGS than in patients with MCD (38.1 vs. 9.5%, *p* < 0.01). So, the patients with FSGS, initial SRNS, cases of positive family history or those with extra-renal manifestations are more likely to due to the genetic cause. In clinical practice, these patients are suggested to do genetic testing.

This study also had some limitations. First, 84 study participants in the present study were lost to follow-up, Thus, there was no chance to observe the effect of treatment and the renal outcome. Second, because our study is retrospective, it is inevitable that data may be missing and there is possibility for information bias. In addition, it is not feasible to complete multivariate analysis in order to find the factors those relate with the gene variants in SRNS.

In conclusion, the overall mutation detection rate in this cohort of Chinese children with SRNS was 37.5%. WT1 was the most frequently detected mutation, followed by NPHS1, NPHS2, and ADCK4. In the higher frequently ADCK4-related SRNS (12.3% in monogenic SRNS), two mutations, c.737G>A (p.S246N) and c.748G>C (p.D250H), were the most prevalent. The detection rate of ADCK4 was quite higher than the rate from Western country. Our indications for genetic testing are patients with FSGS, initial SRNS, cases of positive family history or those with extra-renal manifestations.

## Data Availability Statement

The raw data supporting the conclusions of this article will be made available by the corresponding authors on reasonable request.

## Ethics Statement

The studies involving human participants were reviewed and approved by Ethics Committees of the 23 centers in this study. Written informed consent to participate in this study was provided by the participants' legal guardian/next of kin.

## Author Contributions

All authors listed have made a substantial, direct, and intellectual contribution to the work and approved it for publication.

## Funding

This study was supported by the National Natural Foundation of China (U20A20351), Key Research and Development Plan of Zhejiang Province (2021C03079), the Major projects jointly constructed by the Zhejiang Province and National Health Commission (WKJ-ZJ-1908), National Twelfth Five-Year Science and Technology Support Project (No. 2012BAI03B02), National Key Research and Development Program of China (2016YFC0901505), and Beijing key laboratory of molecular diagnosis and study on pediatric genetic diseases (BZ0317). These funding sustained the design of the study, and the collection and analysis of data.

## Conflict of Interest

The authors declare that the research was conducted in the absence of any commercial or financial relationships that could be construed as a potential conflict of interest.

## Publisher's Note

All claims expressed in this article are solely those of the authors and do not necessarily represent those of their affiliated organizations, or those of the publisher, the editors and the reviewers. Any product that may be evaluated in this article, or claim that may be made by its manufacturer, is not guaranteed or endorsed by the publisher.

## References

[1] Noone DG Iijima K Parekh R. Idiopathic nephrotic syndrome in children. Lancet. (2018) 392:61–74. 10.1016/S0140-6736(18)30536-1 29910038

[2] Lee JM Kronbichler A Shin JI Oh J. Current understandings in treating children with steroid-resistant nephrotic syndrome. Pediatr Nephrol. (2021) 36:747–61. 10.1007/s00467-020-04476-9 32086590 PMC7910243

[3] Trautmann A Schnaidt S Lipska-Zietkiewicz BS Bodria M Ozaltin F Emma F. Long-term outcome of steroid-resistant nephrotic syndrome in children. J Am Soc Nephrol. (2017) 28:3055–65. 10.1681/ASN.2016101121 28566477 PMC5619960

[4] Lombel RM Gipson DS Hodson EM. Treatment of steroid-sensitive nephrotic syndrome: new guidelines from KDIGO. Pediatr Nephrol. (2013) 28:415–26. 10.1007/s00467-012-2310-x 23052651

[5] Tullus K Webb H Bagga A. Management of steroid-resistant nephrotic syndrome in children and adolescents. Lancet Child Adolesc Health. (2018) 2:880–90. 10.1016/S2352-4642(18)30283-9 30342869

[6] Kemper MJ Lemke A. Treatment of genetic forms of nephrotic syndrome. Front Pediatr. (2018) 6:72. 10.3389/fped.2018.00072 29632851 PMC5879576

[7] McCarthy HJ Saleem MA. Genetics in clinical practice: nephrotic and proteinuric syndromes. Nephron Exp Nephrol. (2011) 118:e1–8. 10.1159/000320878 21071976

[8] Preston R Stuart HM Lennon R. Genetic testing in steroid-resistant nephrotic syndrome: why, who, when and how? Pediatr Nephrol. (2019) 34:195–210. 10.1007/s00467-017-3838-6 29181713 PMC6311200

[9] Atmaca M Gülhan B Atayar E Bayazit AK Candan C Arici M. Long-term follow-up results of patients with ADCK4 mutations who have been diagnosed in the asymptomatic period: effects of early initiation of CoQ10 supplementation. Turk J Pediatr. (2019) 61:657–63. 10.24953/turkjped.2019.05.003 32104996

[10] Atmaca M Gulhan B Korkmaz E Inozu M Soylemezoglu O Candan C. Follow-up results of patients with ADCK4 mutations and the efficacy of CoQ10 treatment. Pediatr Nephrol. (2017) 32:1369–75. 10.1007/s00467-017-3634-3 28337616

[11] Lovric S Ashraf S Tan W Hildebrandt F. Genetic testing in steroid-resistant nephrotic syndrome: when and how? Nephrol Dial Transplant. (2016) 31:1802–13. 10.1093/ndt/gfv355 26507970 PMC6367944

[12] Sadowski CE Lovric S Ashraf S Pabst WL Gee HY Kohl S. A single-gene cause in 295% of cases of steroid-resistant nephrotic syndrome. J Am Soc Nephrol. (2015) 26:1279–89. 10.1681/ASN.2014050489 25349199 PMC4446877

[13] Wang F Zhang Y Mao J Yu Z Yi Z Yu L. Spectrum of mutations in Chinese children with steroid-resistant nephrotic syndrome. Pediatr Nephrol. (2017) 32:1181–92. 10.1007/s00467-017-3590-y 28204945 PMC5478193

[14] Trautmann A Vivarelli M Samuel S Gipson D Sinha A Schaefer F. IPNA clinical practice recommendations for the diagnosis and management of children with steroid-resistant nephrotic syndrome. Pediatr Nephrol. (2020) 35:1529–61. 10.1007/s00467-020-04519-1 32382828 PMC7316686

[15] Park E Lee C Kim NKD Ahn YH Park YS Lee JH. Genetic study in Korean pediatric patients with steroid-resistant nephrotic syndrome or focal segmental glomerulosclerosis. J Clinic Med. (2020) 9:2013. 10.3390/jcm9062013 32604935 PMC7355646

[16] Ogino D Hashimoto T Hattori M Sugawara N Akioka Y Tamiya G. Analysis of the genes responsible for steroid-resistant nephrotic syndrome and/or focal segmental glomerulosclerosis in Japanese patients by whole-exome sequencing analysis. J Hum Genet. (2016) 61:137–41. 10.1038/jhg.2015.122 26467726

[17] Rao J Liu X Mao J Tang X Shen Q Li G. Genetic spectrum of renal disease for 1001 Chinese children based on a multicenter registration system. Clin Genet. (2019) 96:402–10. 31328266 10.1111/cge.13606

[18] Mekahli D Liutkus A Ranchin B Yu A Bessenay L Girardin E. Long-term outcome of idiopathic steroid-resistant nephrotic syndrome: a multicenter study. Pediatr Nephrol. (2009) 24:1525–32. 10.1007/s00467-009-1138-5 19280229

[19] Björk J Nyman U Larsson A Delanaye P Pottel H. Estimation of the glomerular filtration rate in children and young adults by means of the CKD-EPI equation with age-adjusted creatinine values. Kidney Int. (2021) 99:940–7. 10.1016/j.kint.2020.10.017 33157151

[20] Zhong XH Ding J Zhou JH Yu ZH Sun SZ Bao Y. [A multicenter study of reference intervals for 15 laboratory parameters in Chinese children]. Zhonghua er ke za zhi = *Chin Journal Pediatr*. (2018) 56:835–45. 10.3760/cma.j.issn.0578-1310.2018.11.009 30392208

[21] Bean L Bayrak-Toydemir P. American college of medical genetics and genomics standards and guidelines for clinical genetics laboratories, 2014 edition: technical standards and guidelines for Huntington disease. Genet Med. (2014) 16:e2. 10.1038/gim.2014.146 25356969

[22] Nagano C Yamamura T Horinouchi T Aoto Y Ishiko S Sakakibara N. Comprehensive genetic diagnosis of Japanese patients with severe proteinuria. Sci Rep. (2020) 10:270. 10.1038/s41598-019-57149-5 31937884 PMC6959278

[23] Nagano C Takaoka Y Kamei K Hamada R Ichikawa D Tanaka K. Genotype-phenotype correlation in WT1 Exon 8 to 9 missense variants. Kidney Int Rep. (2021) 6:2114–21. 10.1016/j.ekir.2021.05.009 34386660 PMC8343804

[24] Bérody S Heidet L Gribouval O Harambat J Niaudet P Baudouin V. Treatment and outcome of congenital nephrotic syndrome. Nephrol Dial Transplant. (2019) 34:458–67. 10.1093/ndt/gfy015 29474669

[25] Caridi G Gigante M Ravani P Trivelli A Barbano G Scolari F. Clinical features and long-term outcome of nephrotic syndrome associated with heterozygous NPHS1 and NPHS2 mutations. Clin J Am Soc Nephrol. (2009) 4:1065–72. 10.2215/CJN.03910808 19406966 PMC2689885

[26] Korkmaz E Lipska-Zietkiewicz BS Boyer O Gribouval O Fourrage C Tabatabaei M. ADCK4-Associated glomerulopathy causes adolescence-onset FSGS. J Am Soc Nephrol. (2016) 27:63–8. 10.1681/ASN.2014121240 25967120 PMC4696579

[27] Malakasioti G Iancu D Tullus K. Calcineurin inhibitors in nephrotic syndrome secondary to podocyte gene mutations: a systematic review. Pediatr Nephrol. (2021) 36:1353–64. 10.1007/s00467-020-04695-0 32651716

[28] Faul C Donnelly M Merscher-Gomez S Chang YH Franz S Delfgaauw J. The actin cytoskeleton of kidney podocytes is a direct target of the antiproteinuric effect of cyclosporine A. Nat Med. (2008) 14:931–8. 10.1038/nm.1857 18724379 PMC4109287

[29] Wakamatsu A Fukusumi Y Hasegawa E Tomita M Watanabe T Narita I. Role of calcineurin (CN) in kidney glomerular podocyte: CN inhibitor ameliorated proteinuria by inhibiting the redistribution of CN at the slit diaphragm. Physiol Rep. (2016) 4:e12679. 10.14814/phy2.12679 27009276 PMC4814882

[30] Song X Fang X Tang X Cao Q Zhai Y Chen J. COQ8B nephropathy: Early detection and optimal treatment. Mol Genomic Med. (2020) 8:e1360. 10.1002/mgg3.1360 32543055 PMC7434746

[31] Trautmann A Bodria M Ozaltin F Gheisari A Melk A Azocar M. Spectrum of steroid-resistant and congenital nephrotic syndrome in children: the PodoNet registry cohort. Clinic J Am Soc Nephrol. (2015) 10:592–600. 10.2215/CJN.06260614 25635037 PMC4386250

[32] Malone AF Phelan PJ Hall G Cetincelik U Homstad A Alonso AS. Rare hereditary COL4A3/COL4A4 variants may be mistaken for familial focal segmental glomerulosclerosis. Kidney Int. (2014) 86:1253–9. 10.1038/ki.2014.305 25229338 PMC4245465

[33] Maselli RA Arredondo J Ferns MJ Wollmann RL. Synaptic basal lamina-associated congenital myasthenic syndromes. Ann N Y Acad Sci. (2012) 1275:36–48. 10.1111/j.1749-6632.2012.06807.x 23278576

[34] Mufson EJ He B Ginsberg SD Carper BA Bieler GS Crawford F. Gene profiling of nucleus basalis tau containing neurons in chronic traumatic encephalopathy: a chronic effects of neurotrauma consortium study. J Neurotrauma. (2018) 35:1260–71. 10.1089/neu.2017.5368 29338612 PMC5962931

[35] Becherucci F Landini S Cirillo L Mazzinghi B Romagnani P. Look Alike, Sound Alike: Phenocopies in Steroid-Resistant Nephrotic Syndrome. Int J Environ Res Public Health. (2020) 17:8363. 10.3390/ijerph17228363 33198123 PMC7696007

