# Supplementary material for: The Clinical and Genetic Features in Chinese Children With Steroid-Resistant or Early-Onset Nephrotic Syndrome: A Multicenter Cohort Study
Source: Front Med (Lausanne). 2022 Jun 9;9:885178. doi: 10.3389/fmed.2022.885178 (PMC9218096; doi:10.3389/fmed.2022.885178)
Supplement: Supplementary file 1 [file Data_Sheet_1.PDF]

Supplementary Table 1. Genotypes and phenotypes of patients with disease-causing mutations

Gene	Mutation	Patient-ID	Sex	Age at onset	Response to steroid	Immune theapy	Response to immune theapy	time of Kidney Biopsy after diagnosis	Kidney Biopsy	flollow-up time	renal outcome	death	family history	extrarenal manifestations	ACMG
WT1	c.1391A＞T	SRNS-2	Female	10M	no-steroid	N	/	ND	ND	NA	NA	N	N	Wilsm tumor	Likely Pathogenic
	c.1432+ 5G>A	SRNS-5	Female	6Y3M	Early onset-SRNS	Y	N	7Y2M	MCD	5y5m	CKD	N	N	N	Pathogenic
	c.736C> T	SRNS-6	Male	4Y7M	Early onset-SRNS	N	/	ND	ND	3Y	ESKD	N	N	Wilsm tumor	Pathogenic
	c.T721C	SRNS-7	Female	8M	Early onset-SRNS	N	/	ND	ND	2.5Y	RRT	N	N	N	Pathogenic
	c.754G> T	SRNS-8	Male	23D	no-steroid	N	/	ND	ND	NA	RRT	Y	N	Intestinal lymphangiectasia？	Pathogenic
	c.748C> T<br>c.1384C>T<br>c.13401G>T	SRNS-10	Female	1Y2M	Early onset-SRNS	Y	N	ND	ND	NA	NA	N	N	N	Pathogenic
	c.1432+5G>A	SRNS-36	Female	3Y	Early onset-SRNS	Y	N	9Y	FSGS	11Y	Normal eGFR	N	N	N	Pathogenic
	c.1255A>G	SRNS-41	Male	12Y7M	Early onset-SRNS	Y	N	12Y9M	FSGS	2Y	CKD	N	N	N	Likely Pathogenic
	c.1432+5G>A	SRNS-43	Male	2Y5M	Early onset-SRNS	N	/	3Y7M	DMS	NA	RRT	N	N	Hypospadias	Pathogenic
	c.1265-1G>A	SRNS-44	Male	7Y10M	Early onset-SRNS	N	/	ND	ND	1Y8M	RRT	N	N	Wilsm tumor	Pathogenic
	c. 497G>T	SRNS-52		1M	no-steroid	N	/	ND	ND	NA	NA	NA	NA	N	Likely Pathogenic
	c.1168C>T	SRNS-73	Male	3Y11M	SRNS	Y	N	ND	MPGN	NA	NA	N	N	Pseudohermaphroditism	Pathogenic
	c.1180C>T<br>c.IVS7-32C>A	SRNS-74	Female	1Y11M	SRNS	N	/	ND	ND	2Y	ESKD	Y	Y	Wilsm tumor	Likely Pathogenic
	c.1180C>T<br>c.IVS7-32C>A	SRNS-75	Female	1Y11M	SRNS	N	/	ND	ND	2Y	CKD	Y	Y	N	Likely Pathogenic
	c.IVS8+5G>A<br>c.903A>G<br>c.126C>T	SRNS-76	Male	7M	SRNS	N	/	ND	ND	NA	NA	Y	N	Pseudohermaphroditism	Pathogenic
	c.1180C>T<br>c.IVS7-32C>A	SRNS-77	Female	1Y2M	SRNS	N	/	ND	ND	6M	ESKD	Y	N	Pseudohermaphroditism	Likely Pathogenic
	IVS9+5G>A	SRNS-79	Female	8Y1M	SRNS	N	/	ND	ND	7M	CKD	N	N	N	Pathogenic
	c.1180C>T	SRNS-106	Male	9M	Early onset-SRNS	N	/	ND	ND	NA	NA	NA	N	Hypospadias	Pathogenic
	c.1432+4C>T	SRNS-124	Female	6Y3M	Early onset-SRNS	N	/	6Y8M	FSGS	5M	Normal eGFR	N	N	N	Pathogenic
	c.698C>A	SRNS-136	Female	8Y2M	no-steroid	N	/	8Y2M	FSGS	NA	NA	NA	NA	N	Pathogenic
	c.1432+4C>T	SRNS-137	Female	4Y8M	Early onset-SRNS	Y	N	ND	ND	1Y6M	RRT	NA	N	Renal dysplasia、Renal cyst	Pathogenic
	c.1432+5G>A	SRNS-138	Female	1Y11M	SRNS	Y	N	ND	ND	1Y3M	Normal eGFR	NA	无	N	Pathogenic
	c.1432+1G>A	SRNS-161	Female	1Y2M	Early onset-SRNS	N	/	1Y7M	FSGS	NA	NA	NA	N	N	Pathogenic
	c.1384C>T	SRNS-162	Female	7Y5M	Early onset-SRNS	Y	N	ND	ND	7M	CKD	Y	N	N	Pathogenic
	c.1339+1G>C	SRNS-163	Female	6d	no-steroid	N	/	ND	ND	NA	NA	Y	N	N	Pathogenic
	c.1447+4C>T	SRNS-203	Female	6Y8M	Early onset-SRNS	Y	N	7Y	FSGS	1Y	Normal eGFR	N	N	N	Pathogenic
	c.1447+5G>A	SRNS-205	Female	13Y	Early onset-SRNS	Y	N	13Y	FSGS	1Y	CKD	N	N	N	Pathogenic
	c.1432+5G>A	SRNS-211	Female	7M	Early onset-SRNS	Y	N	1Y	MsPGN	4Y	RRT	N	N	N	Pathogenic
	c.A1418G	SRNS-213	Female	1Y3M	Early onset-SRNS	Y	N	1Y4M	FSGS	NA	RRT	N	N	N	Likely Pathogenic
	c.1 432+5G>A	SRNS-219	Female	3Y	Early onset-SRNS	Y	N	ND	ND	1Y9M	Normal eGFR	N	N	N	Pathogenic
	c.1432+5G>A	SRNS-222	Female	4M	no-steroid	N	/	ND	ND	NA	ESKD	Y	N	N	Pathogenic
	c.1432+5G>A	SRNS-230	Female	2Y	Early onset-SRNS	Y	N	2Y	MsPGN	10Y	RRT	N	N	N	Pathogenic

NPHS1	c.2515delC<br>c.928G> A	SRNS-9	Female	1M13D	no-steroid	Y(tacrolimus)	Y	ND	ND	2Y	Normal eGFR	N	N	N	Pathogenic
	c.1707C >G<br>c.2120G >A	SRNS-13	Male	1M	Early onset-SRNS	N	/	ND	ND	NA	NA	Y	N	Encephalodysplasia, Ectrosyndactylia	Pathogenic
	c.2515delC<br>c.3595-2A>G	SRNS-50	Male	1M23D	no-steroid	N	/	ND	ND	NA	NA	N	N	N	Pathogenic
	c.3250insG	SRNS-72	Male	2M	SRNS	N	/	ND	ND	NA	NA	Y	N	N	Pathogenic
	c.1931-1_1931insT<br>c.928G>A	SRNS-90	Male	1M	SRNS	N	/	ND	ND	NA	NA	Y	N	N	Likely Pathogenic
	c.3325C>T<br>c.3118C>T	SRNS-142	Male	24d	Early onset-SRNS	N	/	ND	ND	1Y	Normal eGFR	Y	N	Inguinal hernia	Pathogenic
	c.361G>A<br>c.3250_3251insG	SRNS-144	Male	4Y	Early onset-SRNS	Y	N	11Y	FSGS	7Y	CKD	N	N	N	Likely Pathogenic
	c.1740G>T<br>c.2042G>A	SRNS-145	Male	10D	no-steroid	N	/	ND	ND	NA	NA	NA	N	Premature infant	Likely Pathogenic
	c.928G>A<br>c.3250dupG	SRNS-146	Male	CNS	no-steroid	N	/	ND	ND	NA	NA	NA	N	N	Likely Pathogenic
	c.3478C＞T<br>c.exon 23-29del	SRNS-179	Male	2M	no-steroid	N	/	ND	ND	5Y	RRT	N	N	Premature infant	Pathogenic
	c.3027C>G<br>c.3478C>T	SRNS-190	Male	19d	no-steroid	N	/	ND	ND	NA	NA	NA	N	N	Pathogenic
	c.3325C>T<br>c.3213delG	SRNS-202	Female	1M	no-steroid	N	/	ND	ND	NA	NA	Y	N	N	Pathogenic
	c.3213delG<br>c.3478C>T	SRNS-214	Male	1D	no-steroid	N	/	ND	ND	NA	NA	NA	N	N	Pathogenic
	c.3478C>T<br>c.2633delA	SRNS-215	Male	3M	no-steroid	N	/	ND	ND	NA	NA	NA	N	N	Pathogenic
	c.C2783A<br>c.139delG	SRNS-216	Male	1D	no-steroid	N	/	ND	ND	6Y5M	Normal eGFR	N	N	N	Pathogenic
	c.928G>A<br>c.2172_2173delTG	SRNS-233	Male	1M	Early onset-SRNS	N	/	6M	MsPGN	11M	Normal eGFR	N	N	N	Likely Pathogenic
	c.2121G>A<br>c.1531C>G	SRNS-38	Female	2M	no-steroid	N	/	ND	ND	NA	NA	Y	N	N	Pathogenic
	c.1439A>C<br>c.1500_1507del	SRNS-218	Female	1M15D	no-steroid	N	/	ND	ND	NA	NA	NA	N	N	Pathogenic
NPHS2	c.211C> T<br>c.542C> T	SRNS-11	Female	2Y9M	Early onset-SRNS	Y	N	2Y9M	FSGS	3Y	RRT	N	N	N	Pathogenic
	c.592G> C<br>c.412C> T	SRNS-12	Female	0D	Early onset-SRNS	Y	N	ND	ND	6Y8M	Normal eGFR	N	N	Malnutrition	Pathogenic
	c. 534+2T>C<br>c.593A>C	SRNS-16	Male	6Y	Early onset-SRNS	Y	N	6Y3M	MCD	2Y11M	RRT	N	N	N	Likely Pathogenic
	c.586C>T	SRNS-19	Female	2Y6M	Early onset-SRNS	Y	N	2Y	MsPGN	4M	Normal eGFR	N	Y	N	Pathogenic
	c.467dupT	SRNS-25	Male	10M	Early onset-SRNS	Y	N	5Y	FSGS	4Y	Normal eGFR	N	N	N	Pathogenic
	c.467dupT	SRNS-51	Male	8M	Early onset-SRNS	N	/	1Y	MsPGN	NA	RRT	N	Y	Malnutrition	Pathogenic
	c.452G>A	SRNS-92	Male	6Y4M	Early onset-SRNS	Y	N	NA	FSGS	5M	Normal eGFR	NA	N	N	Likely Pathogenic
	c.211C>T<br>c.467dupT	SRNS-134	Female	2Y5M	Early onset-SRNS	N	/	ND	ND	5Y	ESKD	NA	Y	N	Pathogenic
	c.467dupT	SRNS-147	Female	9M	Early onset-SRNS	Y	N	ND	ND	1Y	Normal eGFR	N	Y	N	Pathogenic
	c.460_467dupT<br>c.739-2A>C	SRNS-148	Male	1M	no-steroid	Y	N	ND	ND	2Y3M	Normal eGFR	NA	N	N	Pathogenic
	c.738+2T>C<br>c.467_468insT	SRNS-149	Male	2Y	Early onset-SRNS	Y	N	ND	ND	8Y	RRT	N	N	Testicular dysplasia	Pathogenic

	c.890C>T	SRNS-150	Female	15Y7M	Early onset-SRNS	N	/	15Y8M	FSGS	NA	NA	NA	Y	Premature infant	Pathogenic
	c.G388A c.T370C	SRNS-217	Male	3Y1M	Early onset-SRNS	Y	N	3Y2M	FSGS	1Y	Normal eGFR	N	N	N	Pathogenic
	c.T762A c.C211T	SRNS-220	Male	7Y	Early onset-SRNS	Y	N	7.5Y	FSGS	1Y7M	Normal eGFR	N	N	N	Pathogenic
	c.370T>C c.535-1G>T	SRNS-232	Male	1Y	Early onset-SRNS	Y(tacrolimus)	Y	1Y	MsPGN	1Y	Normal eGFR	N	N	N	Pathogenic
ADCK4	c.748G>C	SRNS-40	Female	3Y3M	Early onset-SRNS	N	/	NA	FSGS	2Y	Normal eGFR	N	N	N	Pathogenic
	c.748G>C c.472C>T	SRNS-58	Female	4Y	Early onset-SRNS	N	/	5Y	FSGS	NA	RRT	N	N	N	Pathogenic
	c.748G>C	SRNS-68	Female	2Y3M	SRNS	N	/	ND	ND	NA	RRT	N	N	N	Likely Pathogenic
	c.748G>C	SRNS-71	Female	6Y8M	SRNS	N	/	ND	ND	NA	RRT	N	N	N	Pathogenic
	c.748G>C c.1093C>G	SRNS-78	Female	6Y2M	SRNS	N	/	ND	ND	10M	Normal eGFR	N	Y	N	Pathogenic
	c.748G>C c.893+2T>A	SRNS-80	Male	11M8D	SRNS	N	/	ND	ND	NA	NA	N	N	N	Pathogenic
	c.532C>T c.748G>C	SRNS-81	Female	10Y2M	SRNS	Y	N	ND	FSGS	8M	RRT	N	Y	N	Pathogenic
	c.532C>T c.748G>C	SRNS-87	Male	7Y	SRNS	Y	N	ND	MsPGN	5y	RRT	N	Y	N	Pathogenic
	c.737G>A c.1465C>T	SRNS-91	Male	1Y1M	Early onset-SRNS	N	/	ND	ND	NA	NA	N	N	N	Pathogenic
	c.737G>A	SRNS-139	Female	8Y2M	Early onset-SRNS	N	/	9Y	FSGS	1Y	Normal eGFR	NA	Y	N	Pathogenic
	c.748G>C c.448C>T	SRNS-140	Female	8Y	Early onset-SRNS	N	/	10Y9M	FSGS	2Y4M	ESKD	NA	Y	N	Pathogenic
	c.737G>A	SRNS-204	Male	1Y5M	Early onset-SRNS	N	/	ND	ND	2Y7M	NA	N	N	N	Pathogenic
	c.748G>C	SRNS-126	Female	10d	Early onset-SRNS	N	/	ND	ND	NA	NA	NA	Y	N	Likely Pathogenic
TRPC6	c.326G>A	SRNS-42	Female	6Y1M	Early onset-SRNS	Y	N	ND	ND	NA	RRT	N	N	N	Pathogenic
	c.325G>A	SRNS-122	Male	2Y7M	Early onset-SRNS	Y	N	2Y11M	FSGS	NA	RRT	N	N	N	Pathogenic
	c.523C>T	SRNS-130	Male	4M	Early onset-SRNS	N	/	1Y	FSGS	1Y	Normal eGFR	NA	N	N	Likely Pathogenic
	c.523C>T	SRNS-158	Male	2Y	Early onset-SRNS	Y	N	2Y2M	FSGS	1Y5M	RRT	N	N	N	Likely Pathogenic
	c.G2684T	SRNS-212	Male	4Y	Early onset-SRNS	Y	N	4Y2M	Glomerulonephritis	NA	RRT	NA	N	N	Pathogenic
	c.523C>T	SRNS-225	Female	6Y	Early onset-SRNS	Y	N	6Y	FSGS	2Y	RRT	N	N	N	Likely Pathogenic
LAMB2	c.47G>A c.2242C>T	SRNS-100	Female	At birth	no-steroid	N	/	10D	FSGS	NA	NA	NA	N	Premature infant, Pinhead pupil, cataract	Pathogenic
	c.2044_2045insT T	SRNS-132	Female	19d	no-steroid	N	/	ND	ND	NA	NA	NA		Premature infant	Pathogenic
	c.1129T>C c.1888C>T	SRNS-141	Female	8M	Early onset-SRNS	Y	N	ND	ND	4M	CKD	N	N	N	Likely Pathogenic
SMARCAL1	c.2423C>G	SRNS-113	Male	3Y2M	Early onset-SRNS	Y	N	4Y	FSGS	8M	Normal eGFR	NA	N	Premature infant	Likely Pathogenic
	c.25C>T c.293G>A	SRNS-210	Male	7Y	Early onset-SRNS	N	/	ND	ND	2Y	NA	N	N	Short stature, weight loss, skin pigmentation spots, spinal epiphyseal dysplasia, cellular immune deficiency	Pathogenic
ACTN4	c.175T>C	SRNS-226	Female	7Y	Early onset-SRNS	Y	N	7Y	FSGS	3Y	ESKD	N	N	N	Pathogenic
	c.785C>T	SRNS-206	Male	4Y5M	Early onset-SRNS	N	/	ND	ND	NA	NA	Y	N	N	Pathogenic
COL4A5	c.349_360delinsT TGC	SRNS-32	Female	3Y	Early onset-SRNS	Y	N	6Y	FSGS	10Y	Normal eGFR	N	N	N	Pathogenic
	c.2224delC	SRNS-207	Male	9Y2M	Early onset-SRNS	Y	N	9Y	MCD	NA	NA	N	N	N	Pathogenic
	c.868delG	SRNS-209	Male	9Y10M	Early onset-SRNS	Y	N	10Y	FSGS	3Y	CKD	N	N	N	Pathogenic

	c.3622G>T	SRNS-224	Male	8Y	Early onset-SRNS	Y	N	8Y、10Y	FSGS	5Y	Normal eGFR	N	N	N	Pathogenic
	c.1963delC	SRNS-201	Female	8Y	Early onset-SRNS	Y	N	11Y	FSGS	6Y	CKD	N	N	N	Pathogenic
PAX2	c.70dupG	SRNS-46	Female	3Y12M	Early onset-SRNS	Y(CsA、MMF	N	4Y5M	FSGS	3Y	Normal eGFR	N	Y	N	Pathogenic
CLCN5	c.1942C>T	SRNS-83	Male	10Y2M	SRNS	N	/	ND	MCD	NA	NA	N	N	N	Pathogenic
ERCC6	c.1834C >T<br>c.2827C >T	SRNS-30	Male	1Y	Early onset-SRNS	Y	N	ND	ND	2Y	Normal eGFR	N	N	N	Pathogenic
ACTN4	c.776C>A	SRNS-53	Female	6Y	Early onset-SRNS	Y	N	7Y	FSGS	3Y	RRT	N	N	N	Pathogenic
APOE	c.127C>T	SRNS-57	Male	8Y	late onset SRNS	N	/	8Y	protein glomeruloneph	2Y	Normal eGFR	N	N	Hypertension	Pathogenic
INF2	c.228 _c.248<br>delGCTGCTGGA<br>GGCGCTGGCGC	SRNS-82	Female	7Y2M	SRNS	N	/	ND	ND	NA	RRT	N	N	N	Pathogenic
NUP160	c.C3517T<br>c.G2407A	SRNS-85	Female	7Y	SRNS	Y	N	7Y,10Y	FSGS	10Y	RRT	N	Y	N	Likely Pathogenic
NUP107	c.553-1G>C<br>c.2437G>A	SRNS-152	Male	4Y1M	Early onset-SRNS	Y	N	4Y7M	FSGS	1Y8M	Normal eGFR	NA	N	N	Pathogenic
NUP93	c.1537+1G>A<br>c.1472A>T	SRNS-153	Female	1Y1M	Early onset-SRNS	Y	N	1Y2M	FSGS	4M	RRT	N	N	N	Pathogenic
PLCE1	c.4301G>A<br>c.4852G>T	SRNS-154	Male	7M	Early onset-SRNS	Y	N	1Y3M	FSGS	4M	ESKD	Y	NA	Lag of motor development	Likely Pathogenic

NA, not available; ND, not done; CKD, chronic kidney disease; ESKD, end-stage renal disease; eGFR, estimated glomerular filtration rate; MCD, minimal change disease; FSGS, focal segmental glomerulosclerosis; DMS, diffuse mesangial sclerosis; MsPGN, mesangial proliferative glomerulonephritis; Y, y no;RRT, Renal replacement therapy(hemodialysis, peritoneal dialysis, kidney transplantation).
